# A Metagenomic Approach Identified a Novel Phasi Charoen-Like Virus Coinfecting a Chikungunya Virus-Infected Aedes aegypti Mosquito in Brazil

**DOI:** 10.1128/MRA.01572-19

**Published:** 2020-07-30

**Authors:** Marielton dos Passos Cunha, Rafaella Sayuri Ioshino, André Luis Costa-da-Silva, Vivian Petersen, Margareth Lara Capurro, Paolo Marinho de Andrade Zanotto

**Affiliations:** a Department of Microbiology, Institute of Biomedical Sciences, University of Sao Paulo, Sao Paulo, Brazil; b Department of Parasitology, Institute of Biomedical Sciences, University of Sao Paulo, Sao Paulo, Brazil; Queens College

## Abstract

Insect-specific viruses do not replicate in vertebrates. Here, we report the genome sequence of a novel strain of a Phasi Charoen-like virus (PCLV) that was isolated from a wild Aedes aegypti mosquito collected in Aracajú, Sergipe State, Brazil. The coding-complete genome of the PCLV is described in this report.

## ANNOUNCEMENT

The Aedes aegypti mosquito is the main vector of several arboviruses. Although this species can be infected by viruses that circulate among invertebrate and vertebrate hosts, finding an infected wild mosquito is frequently challenging. Studies of the mosquito microbiome using metagenomics led to the discovery of a growing number of insect-specific viruses (ISVs) (1, 2). ISVs naturally infect and replicate in insects or laboratory insect cells but do not replicate in vertebrates or vertebrate cells (3).

Here, we describe the coding-complete genome of a member of the *Bunyaviridae* family that was obtained from an A. aegypti mosquito, which was naturally infected by chikungunya virus (CHIKV), as part of a surveillance study of arboviruses in urban mosquitoes (4). To identify whether there was any novel virus in urban A. aegypti mosquitoes, we collected mosquitoes in Sergipe State, Brazil, as described previously (4). The RNA was extracted from the abdomen of an A. aegypti female mosquito, which was initially macerated and subsequently subjected to extraction using the QIAamp viral RNA minikit (Qiagen, Valencia, CA, USA), purified (treated with DNase I), and concentrated using the RNA Clean and Concentrator-5 kit (Zymo Research, Irvine, CA, USA). The RNA was subjected to next-generation genomic sequencing. Briefly, paired-end RNA libraries were constructed using the TruSeq stranded total RNA HT sample preparation kit, and sequencing was performed using the Illumina NextSeq platform.

**FIG 1:**
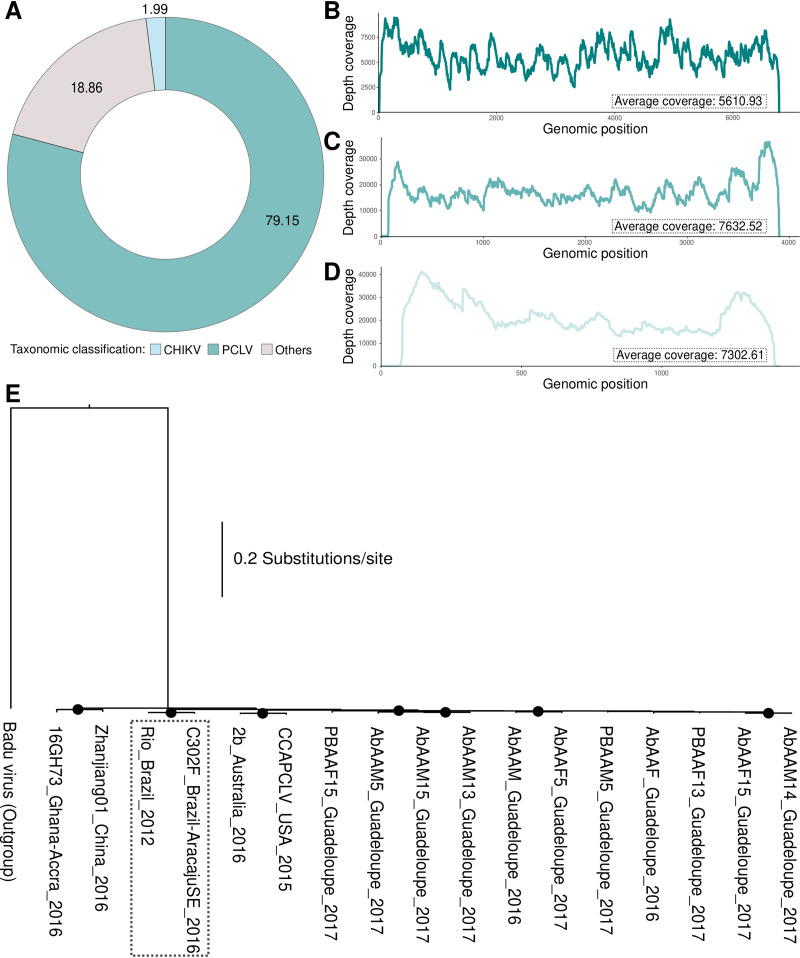
Taxonomic characterization of a novel PCLV. (A) Taxonomic classification of all paired-end reads classified for the mosquito. Short unpaired reads and low-quality bases and reads were removed using Trimmomatic version 0.39 (leading, 20; trailing, 20; slidingwindow, 4:25; minlen, 36). The taxonomic classification of each paired-end read was performed using the Kaiju version 1.7.2 program, based on the reference sequence database provided by the program (completely assembled and annotated reference genomes of archaea, bacteria, and viruses from the NCBI RefSeq database); 79.15% of reads were PCLV, 1.99% were classified as CHIKV, and the rest were classified as reads compatible with other taxa. (B to D) Paired-end reads were assembled *de novo* with SPAdes version 3.13.1 (meta). The taxonomic classification of each assembled contig was determined using the Kaiju version 1.7.2 program, based on the reference sequence database provided by the program. In a second step, we use the three contigs classified as PCLV segments L, M, and S to map to the reference (PCLV contigs) using the program Bowtie 2 version 2.4.1 to determine the average coverage and the coverage for each nucleotide site along in the genome. Shown are the average coverage and the depth coverage for the three PCLV segments, segment L (B), segment M (C), and segment S (D). (E) Phylogenetic characterization of the PCLV based on amino acid sequences (aligned using MUSCLE version 3.8.1551 with default parameters) of all of the concatenated segments (concatenated using a supermatrix approach) of the virus, using a maximum likelihood approach implemented in IQ-TREE version 1.6.12, with the inclusion of other PCLV genomic sequences available in GenBank (up to May 2020, using PCLV sequences that contain all segments and that have the location and year of isolation). To root the tree, we used the reference genome of another phasivirus, the Badu virus (segment L, GenBank accession number NC_038257; segment M, NC_038258; segment S, NC_038259). The black dots indicate bootstrap values above 70. Briefly, the results indicate that the characterized sequences clustered with a virus previously isolated from a mosquito in Brazil (strain Rio) and that all of the Guadeloupe sequences were aggregated into a single group.

Taxonomic analysis of the reads revealed the presence of a large number of reads classified as Phasi Charoen-like virus (PCLV) (79.15%), followed by the presence of CHIKV (1.99%), as previously reported for the same PCLV-positive mosquito (4). All of the other reads were classified as belonging to other viruses and cellular organisms, such as *Archaea* and *Bacteria* (Fig. 1A). Taxonomic analysis of contigs assembled using *de novo* assembly led to the identification of three genome segments of a PCLV (segment L: 6,792 bp; GC content, 38.03%; segment M: 3,907 bp; GC content, 38.52%; segment S: 1,420 bp; GC content, 37.82%), with complete sizes (including coding and noncoding regions) similar to those of the RefSeq PCLV genome and those reported for this virus in the literature (segment L, GenBank accession number NC_038262; segment M, NC_038261; segment S, NC_038263). Genomic coverage was recovered for all sites using a map-to-reference strategy (Fig. 1B to D). Phylogenies indicate that the segments grouped with other PCLV sequences from around the world (1, 2, 5). Interestingly, the viral sequences announced here presented evolutionary relationships closer to that of another Brazilian isolate, which was collected from a mosquito in Brazil in 2012 (strain Rio) (6).

Although PCLV has very often been found in other metagenomic studies (2, 5, 7), we showed that metagenomic investigations may also facilitate understanding of the extant viral diversity in a single mosquito. However, the implications and importance of this PCLV/CHIKV coinfection need to be better studied. Although there are studies that have investigated the modulation of arbovirus infection by ISVs in experimental models, the results are still controversial (8–12). The description presented here will broaden our understanding of the diversity and geographical distribution of insect-specific bunyaviruses and their associations with other viral species.

### Data availability.

Genome data for the PCLV isolate C302F have been deposited in GenBank under the accession numbers MN692603 (segment L), MN692604 (segment M), and MN692605 (segment S). The raw sequencing reads are available in the Sequence Read Archive (SRA) under the accession number PRJNA641154.

## References

[1] Di Giallonardo F, Audsley MD, Shi M, Young PR, McGraw EA, Holmes EC 2018 Complete genome of *Aedes aegypti* anphevirus in the Aag2 mosquito cell line. J Gen Virol 99:832–836. doi:10.1099/jgv.0.001079. 29741476

[2] Zhang X, Jin T, Huang Y, Huang S, Yang F, Lin P, Liu Y, Huang D, Wu C, Xie J, Cheng J, Wan C, Zhang R 2018 Genome sequence of a novel strain of a Phasi Charoen-like virus identified in Zhanjiang. Genome Announc 6:e01024-17. doi:10.1128/genomeA.01024-17. 29326198 PMC5764922

[3] Halbach R, Junglen S, van Rij RP 2017 Mosquito-specific and mosquito-borne viruses: evolution, infection, and host defense. Curr Opin Insect Sci 22:16–27. doi:10.1016/j.cois.2017.05.004. 28805635

[4] Costa-da-Silva AL, Ioshino RS, Petersen V, Lima AF, Cunha M dos P, Wiley MR, Ladner JT, Prieto K, Palacios G, Costa DD, Suesdek L, Zanotto P, Capurro ML 2017 First report of naturally infected *Aedes aegypti* with chikungunya virus genotype ECSA in the Americas. PLoS Negl Trop Dis 11:e0005630. doi:10.1371/journal.pntd.0005630. 28614394 PMC5470658

[5] Zhang X, Huang S, Jin T, Lin P, Huang Y, Wu C, Peng B, Wei L, Chu H, Wang M, Jia Z, Zhang S, Xie J, Cheng J, Wan C, Zhang R 2018 Discovery and high prevalence of Phasi Charoen-like virus in field-captured *Aedes aegypti* in South China. Virology 523:35–40. doi:10.1016/j.virol.2018.07.021. 30077072

[6] Aguiar E, Olmo RP, Paro S, Ferreira FV, De Faria I, Todjro YMH, Lobo FP, Kroon EG, Meignin C, Gatherer D, Imler JL, Marques JT 2015 Sequence-independent characterization of viruses based on the pattern of viral small RNAs produced by the host. Nucleic Acids Res 43:6191–6206. doi:10.1093/nar/gkv587. 26040701 PMC4513865

[7] Shi C, Beller L, Deboutte W, Yinda KC, Delang L, Vega-Rúa A, Failloux A-B, Matthijnssens J 2019 Stable distinct core eukaryotic viromes in different mosquito species from Guadeloupe, using single mosquito viral metagenomics. Microbiome 7:121. doi:10.1186/s40168-019-0734-2. 31462331 PMC6714450

[8] Fredericks AC, Russell TA, Wallace LE, Davidson AD, Fernandez-Sesma A, Maringer K 2019 *Aedes aegypti* (Aag2)-derived clonal mosquito cell lines reveal the effects of pre-existing persistent infection with the insect-specific bunyavirus Phasi Charoen-like virus on arbovirus replication. PLoS Negl Trop Dis 13:e0007346. doi:10.1371/journal.pntd.0007346. 31693659 PMC6860454

[9] Patterson EI, Villinger J, Muthoni JN, Dobel-Ober L, Hughes GL 2020 Exploiting insect-specific viruses as a novel strategy to control vector-borne disease. Curr Opin Insect Sci 39:50–56. doi:10.1016/j.cois.2020.02.005. 32278312 PMC7302987

[10] Kuwata R, Isawa H, Hoshino K, Sasaki T, Kobayashi M, Maeda K, Sawabe K 2015 Analysis of mosquito-borne flavivirus superinfection in *Culex tritaeniorhynchus* (Diptera: Culicidae) cells persistently infected with Culex flavivirus (Flaviviridae). J Med Entomol 52:222–229. doi:10.1093/jme/tju059. 26336307

[11] Romo H, Kenney JL, Blitvich BJ, Brault AC 2018 Restriction of Zika virus infection and transmission in *Aedes aegypti* mediated by an insect-specific flavivirus. Emerg Microbes Infect 7:1–13. doi:10.1038/s41426-018-0180-4. 30429457 PMC6235874

[12] Goenaga S, Kenney JL, Duggal NK, Delorey M, Ebel GD, Zhang B, Levis SC, Enria DA, Brault AC 2015 Potential for co-infection of a mosquito-specific flavivirus, Nhumirim virus, to block West Nile virus transmission in mosquitoes. Viruses 7:5801–5812. doi:10.3390/v7112911. 26569286 PMC4664984

